# Age-Related Measurements of the Myelin Water Fraction derived from 3D multi-echo GRASE reflect Myelin Content of the Cerebral White Matter

**DOI:** 10.1038/s41598-018-33112-8

**Published:** 2018-10-09

**Authors:** Tobias D. Faizy, Dushyant Kumar, Gabriel Broocks, Christian Thaler, Fabian Flottmann, Hannes Leischner, Daniel Kutzner, Simon Hewera, Dominik Dotzauer, Jan-Patrick Stellmann, Ravinder Reddy, Jens Fiehler, Jan Sedlacik, Susanne Gellißen

**Affiliations:** 1 Department of Diagnostic and Interventional Neuroradiology, University Medical Center Hamburg-Eppendorf, Hamburg, Germany; 2 Institute of Neuroimmunology und Multiple Sclerosis, University Medical Center Hamburg-Eppendorf, Hamburg, Germany; 3 Department of Neurology, University Medical Center Hamburg-Eppendorf, Hamburg, Germany; 4 Department of Radiology, University of Pennsylvania, Philadelphia, USA

## Abstract

Myelin Water Fraction (MWF) measurements derived from quantitative Myelin Water Imaging (MWI) may detect demyelinating changes of the cerebral white matter (WM) microstructure. Here, we investigated age-related alterations of the MWF in normal aging brains of healthy volunteers utilizing two fast and clinically feasible 3D gradient and spin echo (GRASE) MWI sequences with 3 mm and 5 mm isotropic voxel size. In 45 healthy subjects (age range: 18–79 years), distinct regions of interest (ROI) were defined in the cerebral WM including corticospinal tracts. For the 3 mm sequence, significant correlations of the mean MWF with age were found for most ROIs (r < −0.8 for WM ROIs; r = −0.55 for splenium of corpus callosum; r = −0.75 for genu of corpus callosum; p < 0.001 for all ROIs). Similar correlations with age were found for the ROIs of the 5 mm sequence. No significant correlations were found for the corticospinal tract and the occipital WM (p > 0.05). Mean MWF values obtained from the 3 mm and 5 mm sequences were strongly comparable. The applied 3D GRASE MWI sequences were found to be sensitive for age-dependent myelin changes of the cerebral WM microstructure. The reported MWF values might be of substantial use as reference for further investigations in patient studies.

## Introduction

Myelin is an important marker for healthy brain function and holds a crucial role in the composition of brain’s microstructure^1^. A microstructural damage of myelin integrity is associated with many functional pathological processes and neurodegenerative diseases of the brain^2^. Therefore, Myelin Water Imaging (MWI) is a powerful quantitative magnetic resonance imaging (MRI) technique for the assessment of the cerebral white matter (WM) microstructure^3^. Myelin water fraction (MWF) has been established to be a potential biomarker for myelin integrity inside the WM^4^. A reduction of the MWF has been reported for several pathologies that affect or damage the pattern of the cerebral WM such as Multiple Sclerosis^4,5^, neuromyelitis optica spectrum disorder^6^, schizophrenia^3^ or chronic stroke^7^.

However, studies investigating age-dependent (demyelinating) changes of the MWF in normal aging brains are rare, albeit normal reference values are mandatory for the future application of MWI sequences in clinical settings and comparative patient studies. Moreover, there is a strong need for pulse sequences, which improve the quality and applicability of MWI in a clinical setting^8^. The necessity to test these fast and “high-end” pulse sequences in a clinical setup has been stated before^8^.

In this study, we utilized two rapid 3D Gradient Echo and Spin Echo (GRASE) sequences, one with 3 mm and one with 5 mm isotropic voxel size, enabling clinically feasible whole brain MWI scans in approximately 15 and 5 minutes, respectively. We aimed to test the technical applicability and performance of both sequences in a clinical setup. Moreover, we sought to generate age-dependent normal values of the MWF in the WM of healthy subjects and also investigated changes of the mean MWF related to age. We hypothesized that the MWF measurements obtained from the 3 mm and 5 mm sequences will be highly comparable and that MWF values measured in normal WM are age-dependent.

## Methods

### Subjects´ characteristics

45 healthy subjects were enrolled in our study (25 male, 20 female). Median age was 36 years (range 18–79 years). The study was approved by the “local research Ethical Committee Hamburg” (“Ethik-Komission der Ärztekammer Hamburg”) following the guidelines of the Declaration of Helsinki and written informed consent was obtained from every subject. All individuals were known to be free of any physical and mental diseases. This was ascertained by standardized questionnaires (attached to the written informed consent form), which included several questions concerning the subjects´ general health condition, as well as questions that evaluated the subjects´ former health history. All forms were reviewed by the supervising physician before any MRI scan was performed. All participants received an MRI examination including two 3D GRASE MWI sequences, T1 weighted (T1w) and fluid attenuated inversion recovery (FLAIR) images. The exclusion of pathological intraparenchymal processes (e.g. tumors, postischemic/posthemorrhage defects etc.) was ensured by surveying the structural MRI scans including 3D T1w and 3D FLAIR images. Leukoaraiosis was considered as a regular process of aging. However, all subjects showed a Fazekas score^9^ of ≤ 1.

### MRI data acquisition

All MRI scans were conducted on a 3 T scanner (Ingenia, Philips Healthcare, Best, the Netherlands) with a 32-channel head and neck coil. The protocol consisted of an axial 3D FLAIR with repetition time (TR) = 4800 ms, echo time (TE) = 289 ms, inversion time (TI) = 1650, Matrix: 224 × 224 × 192, voxel size = 0.5 × 0.5 × 1.1 mm, echo train length (ETL) = 167; a sagittal 3D T1w with TR = 6.4 ms, TE = 2.9 ms, matrix = 256 × 256 × 192, voxel size = 0.94 × 0.94 × 0.94 mm, ETL = 256; two 3D GRASE sequences, which were acquired with 3 mm and 5 mm isotropic voxel sizes with: echo spacing = 6 ms, number of echoes = 32 (maximum TE = 193 ms; TR = 2000ms), ETL = 96; number of slices = 64 slices (3 mm sequence), 32 slices (5 mm sequence); Parallel-imaging = SENSE; SENSE factor = 1.5 was used in both phase encoding directions; Foldover Suppression = No; Over-contiguous slices = No; Number of averages = 1; sagittal orientation; acquisition time: 15 min (3 mm sequence) and 5 min (5 mm sequence). Sagittal slice orientation was used for acquisition to avoid folding-in artefacts in head-foot direction, since non-selective RF-pulses were used.

### Data processing and calculation of MWF maps

MWF maps were extracted from 3D multi echo GRASE data using a novel iterative multi voxel spatial regularization (MVSR) approach^10^, which utilized 3D spatial correlation present in 3D multi echo T2 data as well as effective B1^+^-map to improve on the noise robustness of the reconstruction. As it has been the case traditionally, a slow exchange regime was assumed and thereby, the effect of exchange among various tissue pools (myelin, intra-/extra-cellular water, edema, CSF) was ignored. As pointed out before^10^, utilizing measured B1 + -map would have been suboptimal and hence this processing algorithm accounts for stimulated contributions intrinsically using MET2 data alone. All other data processing steps were identical to the method described by Kumar *et al*.^10^.

### Regions of Interest Analysis

**Figure 1:**
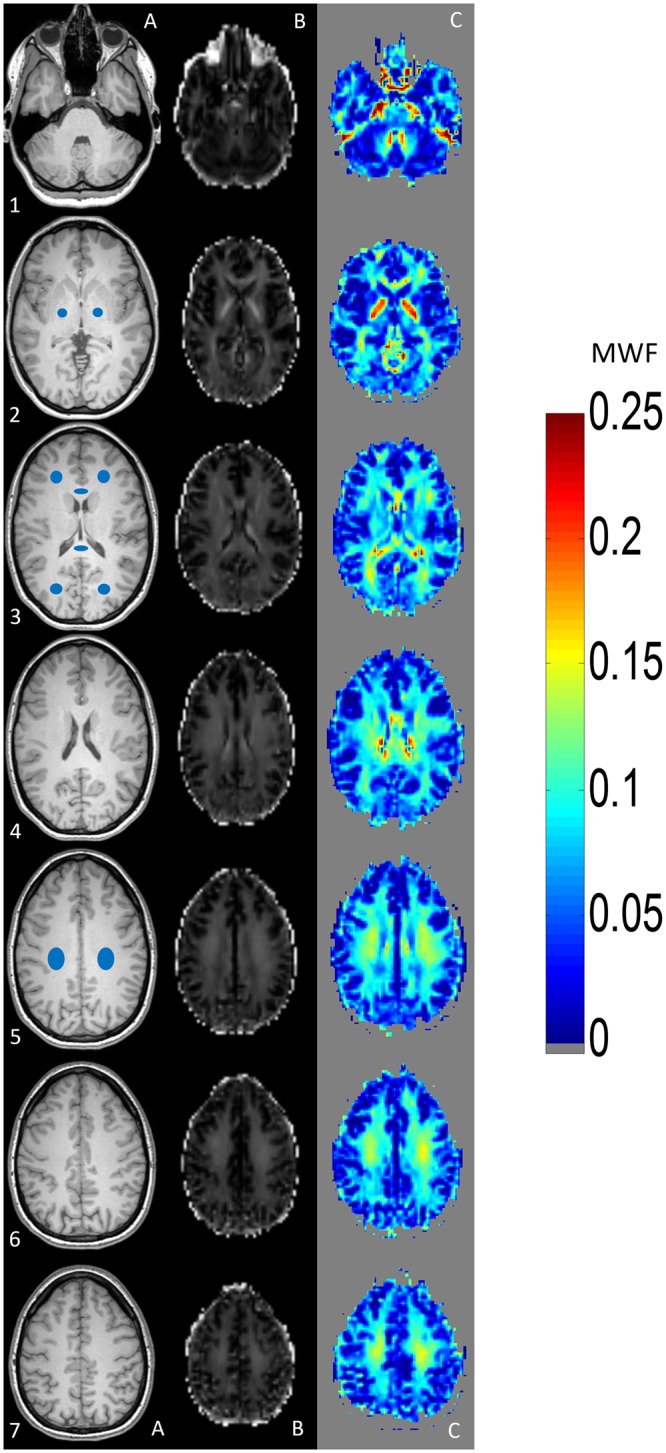
Visualizes a side-by-side comparison between T1-weighted images (left side, column A), the calculated MWF maps (middle, column B) and MWI heat maps (right side, column C). An example for ROI localization is given in column A. The scale on the right side indicates the estimated MWF values. Higher MWF values are indicated by the range of “warm” (red) colours, whereas lower MWF values are indicated by the range of “cold” (blue) colours. ROIs were placed in both corticospinal tracts (section A2), frontal and occipital white matter (WM) and genu- and splenium of corpus callosum (section A3) and the parietal WM of both hemispheres (section A5).

Image data were processed using the software package FSL 5.0 (Analysis Group, FMRIB, Oxford, UK). Both, 3 mm and 5 mm GRASE MWI sequences were acquired consecutively and it was ensured that subjects did not move between the acquisition of the sequences. In addition, the exact same slab position- and orientation was utilized for both MWI sequences. Regions of Interest (ROI) analysis was performed with the software package ANALYZE 11.0 (Analyze Direct, Overland Park, KS, USA). In each subject, 10 ROIs were defined in distinct brain regions. ROIs were outlined in the frontal, parietal and occipital WM and in the corticospinal tract (posterior limb of the internal capsule) on both sides and also in the genu- and splenium of corpus callosum, similar to the ROI localizations described in^4^. For ROI definition, strong T2-weighted images (12^th^ echo of the respective MWI sequence of each subject) were used. Subsequently, predefined ROIs were transferred to the corresponding MWF map (Fig. 1). Due to the different voxel sizes of the 3 mm and 5 mm MWI sequences, accurate and correct ROI positioning was verified by an experienced neuroradiologist with a side-by-side comparison of both MWI sequences. Supplementary Figure 1 provides a visual comparison of calculated MWF maps with 3 mm and 5 mm isotropic voxel sizes from the same subject.

### Statistical analysis

Data are presented as mean ± standard deviation (sd). Statistical significance for the results of hypothesis tests was corrected for multiple testing, if necessary, and assumed with an α error of p ≤ 0.05 or p < 0.001 respectively. Statistical analysis was performed with the software Package SPSS (SPSS 24.0). Normal distribution of the data was tested with the Kolmogorov-Smirnov test. For each ROI, correlations between age and MWF were calculated and described with linear models and Pearson´s correlation coefficients were extracted from these calculations. Bland-Altman plots were used to assess the comparability of mean MWF values measured in 3 mm and 5 mm MWI sequences. Paired two-tailed t-tests were computed to test for side- related differences of the mean MWF in the respective ROIs and to investigate differences of mean MWF values between the 3 mm and 5 mm sequences. Unpaired t-tests were used to test sex-related differences of the mean MWF in the analysed ROIs. Analysis of variances (ANOVA) was used to determine differences of the mean MWF between the distinct ROI localizations.

## Results

### Differences of mean MWF measurements between the right and left hemisphere

**Figure 2:**
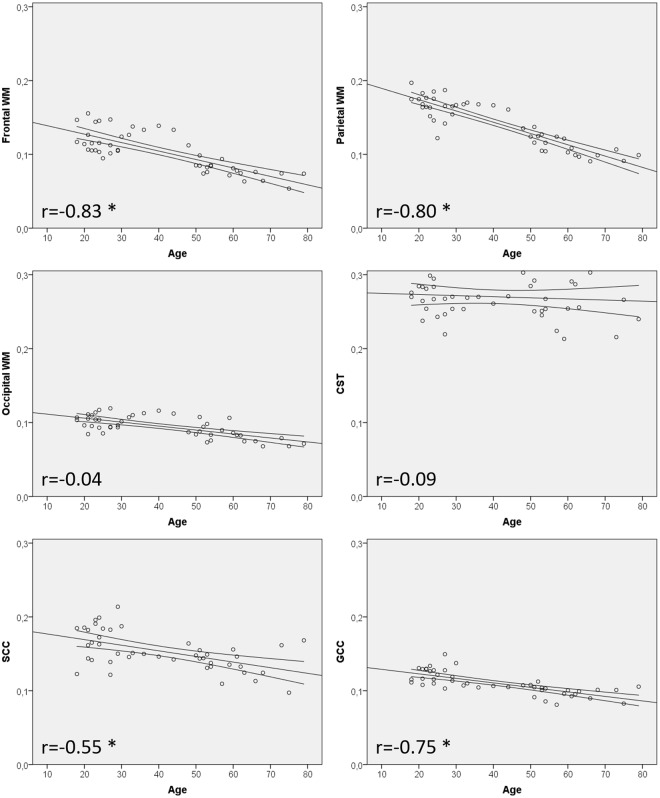
Displays the correlations of mean myelin water fraction (MWF) values and age. Declines of the MWF in the frontal and parietal WM were highly correlated with age. Moderate negative correlations of the MWF with age were found for the genu and splenium of corpus callosum. Upper and lower curved graphs are indicating the 95% confidence interval of the mean value. No significant correlation with age was found for the ROI in the occipital WM and the corticospinal tract. r = correlation coefficient; significant results (p < 0.001) are marked with an asterisk.

**Figure 3:**
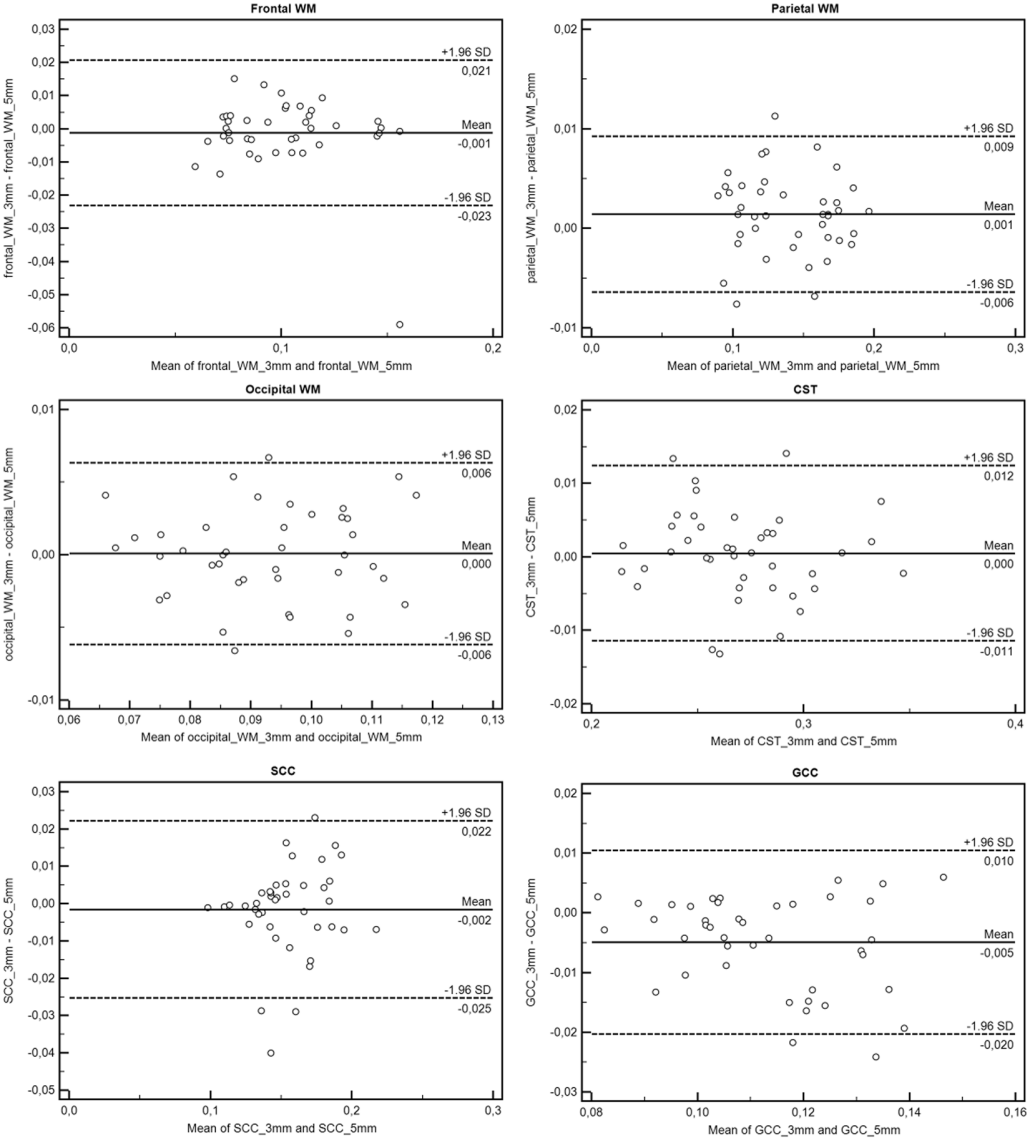
Bland-Altman plots showing the difference between the two MWI sequences (MWI sequence with 3 mm vs 5 mm isotropic voxel size) plotted against their mean in all investigated Regions of Interest (ROI). Evidently, both measurement approaches show a high agreement in terms of the acquired MWF values. WM = white matter, CST = corticospinal tract, SCC = splenium of corpus callosum, GCC = genu of corpus callosum.

In all subjects, the obtained MWF values from both hemispheres were comparable and no significant differences of mean MWF measurements were found between the respective ROIs in the frontal, parietal and occipital WM and CST of both sides (p > 0.05 for all ROIs). Since no significant differences were detected between the hemispheres, mean MWF values of WM and CST-ROIs of both sides were used for Figs 2 and 3. The highest mean MWF values were detected in the CST of both hemispheres. The lowest mean MWF measurements were found in the frontal WM ROIs. Significant differences (p < 0.001) of the mean MWF were found between all ROIs, except for frontal WM vs. occipital WM, frontal WM vs. GCC, parietal WM vs. SCC and occipital WM vs. GCC (p > 0.001).

### Correlations of mean MWF with age

For the 3 mm sequence, our results indicate a strong negative linear correlation of the mean MWF with age in the frontal right (r = −0.823), frontal left (r = −0.842), parietal right (r = −0.831) and parietal left (r = −0.833) WM ROI (p < 0.001 for all ROIs). Moderate negative linear correlations with age were found for the genu of corpus callosum (GCC) (r = −0.746) and splenium of corpus callosum (SCC) (r = −0.546) (p < 0.001 for all ROIs). No significant correlation with age was found for the mean MWF obtained from the occipital WM ROIs and the corticospinal tracts (CST) of both hemispheres. Similar correlations with age were found for the 5 mm sequence in the WM ROIs, the GCC and SCC (r > 0.7, p < 0.001 for all ROIs), with an exception for CST and occipital WM. Figure 2 displays scatter plots of mean MWF values and age for distinct ROI localizations. The equations describing the slope of the regression lines can be used to calculate/estimate mean MWF values for any random year of age in any ROI individually:

3A $$\begin{array}{cc}{\rm{Frontal}}\,\text{WM}: & {\rm{MWF}}=0.15\mbox{--}1.15\times {10}^{-3}\times {\rm{Age}}\end{array}\ldots $$

3B $$\begin{array}{cc}{\rm{Parietal}}\,\text{WM}: & {\rm{MWF}}=0.20\mbox{--}1.53\times {10}^{-3}\times {\rm{Age}}\end{array}\ldots $$

3C $$\begin{array}{cc}{\rm{Occipital}}\,{\rm{WM}}: & {\rm{MWF}}=0.12\mbox{--}5.37\times {10}^{-4}\times {\rm{Age}}\end{array}\ldots $$

3D $$\begin{array}{cc}{\rm{CST}}: & {\rm{MWF}}=0.28\mbox{--}1.5\times {10}^{-4}\times {\rm{Age}}\end{array}\ldots $$

3E $$\begin{array}{cc}\text{SCC}: & {\rm{MWF}}=0.18\mbox{--}7.62\times {10}^{-4}\times {\rm{Age}}\end{array}\ldots $$

3F $$\begin{array}{cc}{\rm{GCC}}: & {\rm{MWF}}=0.14\mbox{--}6.11\times {10}^{-4}\times {\rm{Age}}\end{array}\ldots $$

In addition to simple linear models, we evaluated if a quadratic fit might be more suitable as suggested by others. However, root mean square of fitting errors of the quadratic fitting were always higher than corresponding values for linear fitting, except for the SCC. That justifies fitting linear model to aforementioned MWF vs age trend and we did not report quadratic correlations in this manuscript.

### Sex-dependent differences of the mean MWF

No sex-related differences were detected for the mean MWF for all WM ROIs (p > 0.05 for all ROIs).

### Comparison of mean MWF measurements obtained from 3 mm and 5 mm sequences

Paired-t-test revealed no significant differences of the mean MWF values obtained from the 3 mm and 5 mm sequence in the frontal (p = 0.574), parietal (p = 0.344) and occipital (p = 0.632) WM ROIs, as well as in the CST (p = 0.947), the GCC (p = 0.473) and the SCC (p = 0.565) of both sequences. Bland-Altman-Plots in Fig. 3 display the differences of the MWF measurements obtained from the 3 mm and 5 mm sequence plotted against their averaged means. As evident from the Bland-Altman plot analysis, both measurement approaches (3 mm vs 5 mm isotropic voxel sizes) show a high level of agreement in terms of the acquired MWF values.

## Discussion

Quantitative MRI techniques such as MWI have shown to be sensitive to pathological microstructural changes of brain tissue^3–5,7^. The importance and the necessity to obtain and report normal reference values for quantitative MRI sequences in normal aging healthy brains has firmly been stated before^2,11–13^. The mean MWF measurements obtained in our study are comparable, but somewhat larger, than values reported in other 3D GRASE studies^8,14^ and we observed a similar general trend across all distinct WM ROIs. In our investigation, we found an age-related decline of the mean MWF in most WM ROIs (except occipital WM), in the GCC and SCC, but not in the corticospinal tracts of both hemispheres.

A study by Lang *et al*.^3^ investigated the patterns of aberrant myelination in 58 patients with first-episode of schizophrenia in comparison to 44 healthy volunteers. The authors reported positive correlations of the MWF in early life decades in the frontal WM, the GCC and SCC of healthy volunteers. Although these findings may appear counterintuitive in the first place, the authors claim that these might be attributed to the ongoing physiological process of myelination (and thus increase of the MWF) in early life decades^15^. In comparison to our study, Lang *et al*. only used a single-slice MWI sequence based on a different MWI technique (CPMG sequence) and used different ROI localizations and a different MWF map processing algorithm. In accordance with the findings of Lang *et al*., we also did not detect any significant decreases of the mean MWF with age in most ROIs within the first 4 life decades (Fig. 2), supporting the presumption of ongoing physiological myelination in the first 40 life years.

The findings of our study are in contrast to other age-related imaging studies employing MWI sequences. A cross-sectional imaging study by Billiet *et al*.^16^ investigated age-dependent changes of Multiexponential T2-relaxiation (MET2) measurements (together with diffusion MRI metrics) in normal-aging brains. In contrast to our study, the 3D GRASE sequence utilized in the study by Billiet *et al*. was based on the imaging and MWF map reconstruction approach by Prasloski *et al*.^8^. In all defined ROIs, the authors only found minor differences of MWF metrics with age, implying that MET2 metrics may not be suitable for the evaluation of age-related changes of myelin content. Nevertheless, Billiet *et al*. also found positive correlations of MET2 metrics with age in younger subjects, further supporting the aforementioned concept of ongoing myelination in early life decades.

Another imaging study of Arshad *et al*.^17^ also utilized the Prasloski 3D GRASE MWI sequence in combination with Diffusion Tensor Imaging (DTI) to investigate myelination in distinct subcortical WM tracts in a cohort of 61 healthy volunteers^8,17^. They reported of minor quadratic relationships of MWF measurements in the genu and splenium of corpus callosum with age, whereas we found a moderate negative linear decline of mean MWFs in these ROIs. The diverging findings regarding age-dependent WM changes of MET2 metrics between the related studies (ours vs Billiet *et al*. and Arshad *et al*.) are notable and may be attributed to both experimental and reconstruction factors. On the experimental side, the aforementioned studies^16,17^ utilized longer echo spacing (10 ms for Billiet *et al*. and 11 ms for Arshad *et al*. vs 6 ms for ours), which implies that less echo time-points were available to capture myelin contributions. Moreover, both studies used smaller TR (1000 ms for Billiet *et al*. and 1100 ms for Arshad *et al*. vs 2000 ms in our study), which may have led to significant T1 contamination. On the reconstruction side, the improved quantification accuracy of our method over the method by Prasloski *et al*.^8^, utilized by Billiet *et al*. and Arshad *et al*., has recently been demonstrated by Kumar *et al*.^10^. Other dissimilarities, including different ROI analysis approaches and different scanner models may also have contributed to the different results between our studies.

However, interestingly, Arshad and colleagues found the highest age-related quadratic relationship for the MWF in the CST (anterior and posterior limb of internal capsule), with an increase of MWF values until mid-age, followed by a minor decrease of the MWF from the 6^th^ age-decade. Although we detected age-dependent declines of the mean MWF in most ROIs, no such difference was found in the CST. The CST is holding special MRI characteristics that must be taken into consideration when interpreting its results^18^. Russel-Schultz *et al*. reported that the T_2_ distribution from the CST had an intra/extracellular water peak which was not only shifted towards longer T2 times but also exhibited a second peak with a shorter T_2_ time^18^. The authors concluded that higher MWF values usually found in the CST, might be explained by increased amounts of extracellular water other than increased myelin density of CST fibres. Therefore, age-related associations of MWF estimates in the CST, as described by Arshad *et al*., might also be explained by changes of water-like-components in the vicinity of CST fibres, rather than caused by an equilibrium of myelination and demyelination during aging^18–23^. However, an anterior-posterior gradient has been reported by several imaging studies using quantitative MRI parameters such as FA or MD^24,25^. These studies suggest that microstructural changes with age are greatest in anterior brain regions, while posterior brain regions are well preserved^26^. These reports are in line with our findings, since we did not detect any age-related declines of the mean MWF in the occipital WM, but in frontal located brain areas.

Findings of advanced MRI imaging studies indicate a degeneration of the cerebral WM microstructure with age, presumably based on a loss of axons, various degrees of demyelination, disrupted macrostructural organization, concomitant with an enlargement of the extracellular space^16,25,27^. These findings are reflected by histopathological reports that normal brain aging is marked by degeneration of WM including myelin pallor^28^, loss of myelinated nerve fibres^29,30^ and malformation of myelin sheaths^11,31^. It is conceivable that a certain portion of these age-related demyelinating processes might be reflected by a reduction of the MWF. However, the physiology of age-related changes in the cerebral WM microstructure is a complex and heterogeneous process that is associated with a high degree of inter-individual variability^11^. More extended cross-sectional studies, or even better longitudinal investigations, would be needed to particularly elucidate the underlying processes that led to the here reported age-dependent declines of the mean MWF in healthy subjects.

We also acquired data using a GRASE MWI sequence with an isotropic voxel size of 5 mm (5 min). This rapid sequence provides whole brain MWI in reasonable acquisition times, improving the applicability for the clinical routine. Our findings (Fig. 3) indicate that mean MWF values derived from the 3 mm and 5 mm sequence are strongly consistent with each other. Several studies have pointed out the necessity to evaluate (early) changes of the WM microstructure, particularly in order to detect indices for demyelination even before signal abnormalities are visible on conventional MRI scans^2,4,5,32^. Thus, a fast and reliable pulse sequence with clinically feasible acquisition times might be able to serve for the early detection of abnormalities in the normal appearing white matter (NAWM) of patients with e.g. MS or clinically isolated syndrome, which can be mandatory for a timely therapy-onset or to rule out other differential diagnosis.

Our study holds some limitations. Although we applied the same slab-orientation and positioning for the acquisition of the 3 mm and 5 mm 3D GRASE sequences, we cannot exclude that in rare cases, ROI localizations may differ minimally between the sequences due to partial volume effects. However, the 5 mm sequence used in our study still suffers from these partial volume effects and a low contrast-to-noise ratio (CNR), which is a common finding in many (low-resolution) MWI sequences (discrimination between grey and white matter, tough differentiability of brain tissue at the CSF boundary)^4,33^, as well as a considerably low spatial resolution, which is however sufficient for measurements in larger structures such as the WM. For the time being, the 5 mm sequence might be suitable to detect regionally selective WM changes throughout the brain but may be less efficient to detect focal WM abnormalities inside of very small pathologies or at the border of WM/CSF. More extended (longitudinal) study designs including greater subject numbers are needed to explicitly investigate age-dependent alterations of the MWF in the particular life-decades. Due to the small sample size of our study cohort, the here presented MWF values are only of descriptive nature, albeit a trend of decreasing mean MWF values with age was evident.

## Conclusion

The conducted 3D GRASE MWI sequence seems to be a sensitive and versatile tool for the detection of age-dependent changes of the cerebral white matter microstructure, presumably caused by demyelination. The provided reference values obtained from healthy subjects might be of substantial use for further investigations of pathological WM changes in patient studies using MWI techniques.

## Electronic supplementary material

Supplementary Figure

Supplementary Table

## Data Availability

All data generated or analysed during this study are included in this published article (and its Supplementary Information files).
