# Supplementary material for: Age-Related Measurements of the Myelin Water Fraction derived from 3D multi-echo GRASE reflect Myelin Content of the Cerebral White Matter
Source: Sci Rep. 2018 Oct 9;8:14991. doi: 10.1038/s41598-018-33112-8 (PMC6177453; doi:10.1038/s41598-018-33112-8)
Supplement: Supplementary file 1 — Supplementary Figure [file 41598_2018_33112_MOESM1_ESM.docx]

Age-Related Measurements of the Myelin Water Fraction derived from 3D multi-echo GRASE reflect Myelin Content of the Cerebral White Matter

Dr. Tobias D. Faizy^1^, Dr. Dushyant Kumar1, Dr. Gabriel Broocks^1^, Dr. Christian Thaler^1^, Dr. Fabian Flottmann^1^, Dr. Hannes Leischner^1^, Daniel Kutzner^1^, Simon Hewera^1^, Dominik Dotzauer^1^, Dr. Jan-Patrick Stellmann^2,3^, Dr. Ravinder Reddy^4^, Prof. Dr. Jens Fiehler^1^, Dr. Jan Sedlacik^1^*, PD Dr. Susanne Gellißen^1^*

^1^Department of Diagnostic and Interventional Neuroradiology, University Medical Center Hamburg-Eppendorf, Hamburg, Germany.

^2^ Institute of Neuroimmunology und Multiple Sclerosis, University Medical Center Hamburg-Eppendorf, Hamburg, Germany

^3^Department of Neurology, University Medical Center Hamburg-Eppendorf, Hamburg, Germany

^4^ Department of Radiology, University of Pennsylvania, Philadelphia, USA

*The authors Sedlacik and Gellißen contributed equally to this study

**University Medical Center Hamburg-Eppendorf**

**Martinistrasse 52**

**20246 Hamburg-Eppendorf**

**Germany**

**E-mail:** [**T.Faizy@uke.de**](mailto:T.Faizy@uke.de)

**Phone: 0049-152-228 16 512**

**Supplementary Information**

Supplementary Figures

**Supplementary Figure 1** Comparison of Myelin Water Fraction maps calculated with 3mm and 5mm isotropic voxel size


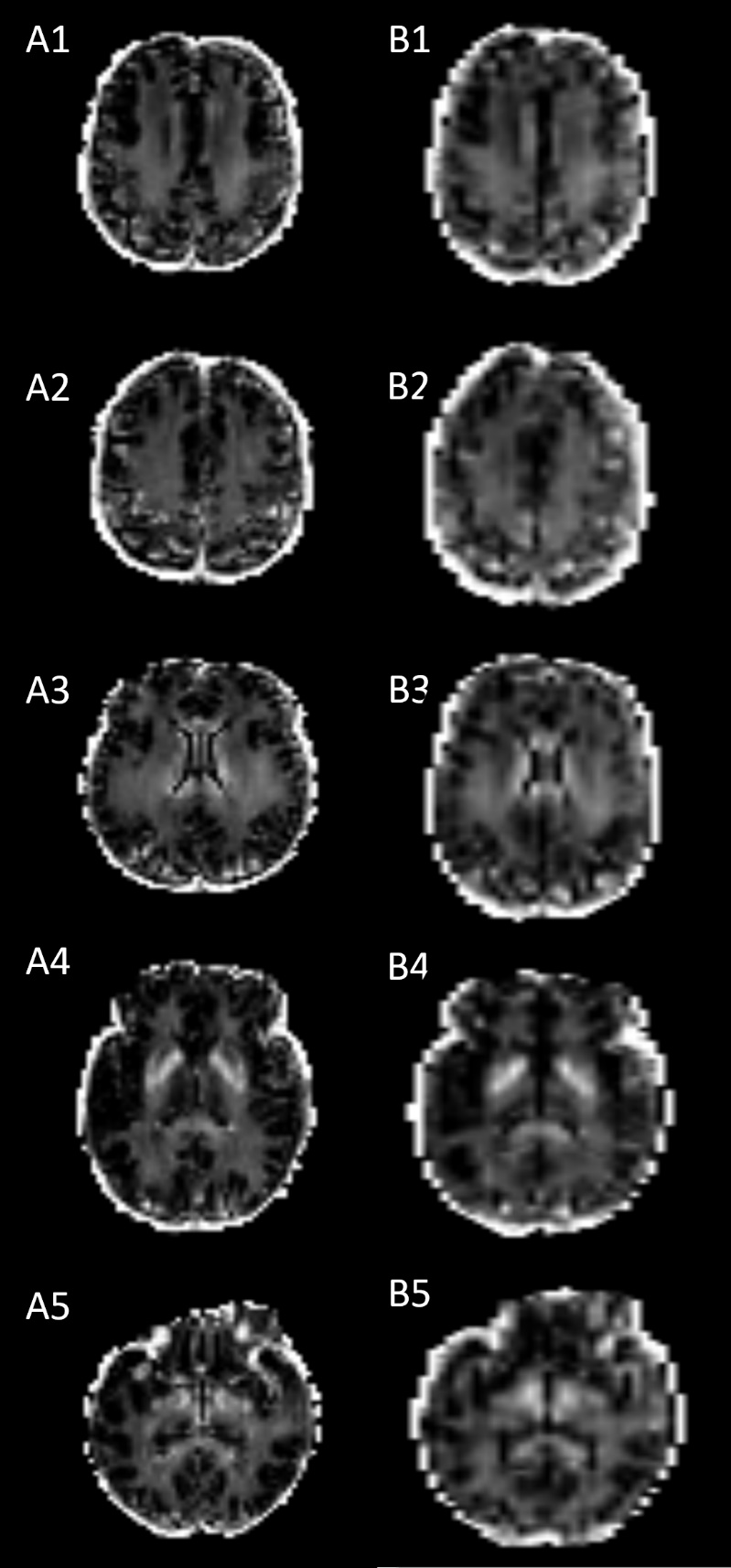


Supplementary Figure 1 displays a visual comparison of calculated MWF maps with 3mm (A1-A5) and 5mm (B1-B5) isotropic voxel sizes of a 21-year old healthy volunteer.
